# The impact of self-management ability on oral frailty in older adults with hypertension: the chain mediating role of anxiety and nutritional status

**DOI:** 10.3389/fmed.2026.1796196

**Published:** 2026-06-12

**Authors:** Min Xu, Xu Zhang, Chernor Sulaiman Bah, Yue Yuan, Yan Wang, Chenjian Zhao, Changke Ma, Zexiang Bao

**Affiliations:** 1 Office of Medical Consortium Administration, Nanjing Luhe People’s Hospital, Yangzhou University, Nanjing, China; 2 School of Nursing and School of Public Health, Yangzhou University, Yangzhou, China; 3 Department of Information, Luhe District Hospital of Traditional Chinese Medicine, Nanjing, China; 4 School of Basic Medicine and Clinical Pharmacy, China Pharmaceutical University, Nanjing, China; 5 Department of Stomatology, Nanjing Luhe District People's Hospital, Nanjing, China; 6 Department of Orthopedics, Nanjing Luhe People’s Hospital, Nanjing, China; 7 Department of Pharmacy, Luhe District People's Hospital, Nanjing, China; 8 Department of Pharmacy, Nanjing First Hospital, China Pharmaceutical University, Nanjing, China

**Keywords:** anxiety, chainmediation, nonlinear relationship, nutritional status, older adults with hypertension, oral frailty, theory of unpleasant symptoms

## Abstract

**Background:**

The incidence of oral frailty among older hypertensive adults is as high as 61.5%, which is mainly characterized by impairments in oral functions such as chewing and speech, which in turn increases the risk of adverse outcomes, and mortality. Previous studies have shown that hypertension self-management ability, anxiety, and nutritional status are influencing factors of oral frailty, yet the mechanisms underlying their interactions remain unclear.

**Methods:**

This study adopted a cross-sectional survey design. Using convenience sampling, 740 inpatients with hypertension were recruited from five medical institutions in Jiangsu Province. Data were collected through structured face-to-face interviews using the following scales: Self-Management Scale for Patients with Hypertension (SMSPH), Generalized Anxiety Disorder 7-item Scale (GAD-7), Short-Form Mini-Nutritional Assessment (MNA-SF), and Oral Frailty Index-8 (OFI-8). Spearman’s correlation analysis was used to test the pairwise correlations among variables, and structural equation modeling (SEM) was employed to analyze mediating effects.

**Results:**

There were significant pairwise correlations between hypertension self-management ability, oral frailty, anxiety, and nutritional status (*p* < 0.001). Self-management ability not only exerted a direct negative effect on oral frailty (*β* = −0.037, 95% CI: −0.054 to −0.019) but also acted through three indirect pathways: the anxiety pathway (*β* = −0.017, 95% CI: −0.024 to −0.011), the nutritional status pathway (*β* = −0.008, 95% CI: −0.014 to −0.004), and the serial mediation pathway of anxiety-nutritional status (*β* = −0.009, 95% CI: −0.019 to −0.003). The total mediating effect accounted for 47.90% of the total effect.

**Conclusion:**

Anxiety and nutritional status play mediating roles in the relationship between hypertension self-management ability and oral frailty in older hypertensive adults, with a serial mediating effect of anxiety-nutritional status. Clinicians could implement comprehensive intervention measures to improve patients’ self-management ability, alleviate anxiety, and optimize nutritional status, thereby preventing and controlling oral frailty.

## 1 Introduction

Hypertension is a major public health issue threatening the health of the elderly worldwide. As a high-risk group for oral health problems, patients with hypertension have higher rates of dental caries, reduced salivary secretion, and periodontal disease compared to healthy individuals (1). In recent years, such oral health issues have been summarized as Oral Frailty (OF), a concept that has gained increasing global attention as it not only covers individual indicators of oral diseases but also comprehensively reflects cumulative deficiencies in oral health (2). The incidence of oral frailty in rural older hypertensive patients was 61.5% (3). OF is reversible in its early stages and can be improved through personalized interventions (4). In addition, early intervention for this high-risk group of older adults with hypertension can further prevent deterioration of OF. It is of great significance to delay or reverse the progression of oral frailty and improve their quality of life. To develop effective prevention and treatment strategies, it is urgently necessary to explore its underlying mechanisms in depth.

However, existing studies predominantly focus on the prevalence of OF and the analysis of influencing factors (5, 6), lacking an integrative theoretical model to systematically explore multiple factors. Therefore, to deeply analyze the multi-factor interaction mechanism, this study introduces the “Theory of Unpleasant Symptoms” (TOUS). This theory posits that unpleasant symptoms (oral frailty) arise from the combined effects of physiological, psychological, and situational factors, with complex interaction mechanisms among these factors rather than simple independent influences (7).

Within the overarching framework of TOUS, hypertension self-management ability (HSMA) is recognized as a contextual factor influencing dysphoria symptoms. This encompasses comprehensive management measures such as regular medication adherence, balanced diet, consistent physical activity, and emotional monitoring (8, 9). Notably, individuals with low self-management levels exhibit unhealthy lifestyles, including smoking, a high-carbohydrate and high-fat diet, and insufficient intake of fruits and vegetables. These factors are not only risk factors for cardiovascular diseases but also have been confirmed as susceptibility factors for periodontal disease (10). Therefore, active participation in self-management may delay the progression of hypertension and alleviate symptoms (11, 12). Additionally, it may directly protect the oral microenvironment and tissue structure by promoting coordinated health behaviors such as regular oral hygiene and periodic dental check-ups, thereby delaying the onset and development of oral deterioration. Based on the aforementioned background, this study proposes the following Hypothesis 1(H1): There is a negative correlation between HSMA and OF.

Anxiety (psychological factor) is a multifaceted and ubiquitous emotional state. It is primarily characterized by the manifestation of a constellation of negative emotions, including but not limited to restlessness, nervousness, apprehension, and fear (13). Self-management proficiency is negatively correlated with anxiety levels (14). Inadequate management of hypertension exacerbates patients’ fear and despair of complications, thereby inducing or aggravating anxiety and unease (15). Anxiety-induced chronic psychological stress leads to alterations in the quality and quantity of salivary secretion and a decline in immune defense function, directly increasing the risk of dental caries and periodontal disease (16). Therefore, Hypothesis 2(H2) posits that anxiety plays an independent mediating role between HSMA and OF.

Further research has found that long-term poor control of hypertension induces chronic inflammation in tissues and organs, including blood vessels. This, in turn, accelerates the catabolism of lipids and proteins, thereby increasing the risk of malnutrition (physiological factor) (17). Hypertensive older adults with malnutrition exhibit poorer oral health, have fewer functional teeth, and are more prone to oral soft tissue inflammations such as lip dryness or cracking, gingival edema, and ulcers, accelerating the progression of OF (18, 19). Based on this, the present study proposes the following parallel mediating Hypothesis 3(H3): Nutritional status plays an independent mediating role between HSMA and OF.

TOUS posits that there is a bidirectional interaction between physiological and psychological factors which facilitates a more comprehensive understanding of how HSMA influences OF. Within this framework, HSMA (contextual factor) may first affect anxiety (psychological factor), which in turn influences nutritional status (physiological factor), and ultimately acts on OF (unpleasant symptom). Among older adults, anxiety has been confirmed as a predictor of malnutrition, with a positive correlation between the two (20, 21), which is consistent with the syndrome of “weakness of the heart and spleen” in traditional Chinese medicine. A bad mood can easily induce liver qi stagnation. Qi stagnation causes the obstruction of qi and blood in the body, spleen, and liver, such as spleen insufficiency, affects the digestive system, and leads to a decreased appetite (22). It is evident that anxiety and nutritional status are not only interrelated but may also follow a progressive influence pathway. To explore the specific manifestation of this physio-psychological interaction in the occurrence and development of OF, this study attempts to construct and test a serial mediation model to systematically analyze the potential influence sequence from psychological state (anxiety) to physiological condition (nutritional status). Based on this, Hypothesis 4 (H4) is proposed: HSMA could negatively effect OF through the serial mediation pathway of anxiety and nutritional status.

Based on existing empirical evidence, there is a correlation between HSMA, anxiety, nutritional status, and OF. However, no specific mechanisms have been reported. To address this, based on the interactive relationships among the multidimensional factors within TOUS, this study aims to employ structural equation modeling (SEM) to test the aforementioned hypotheses, thereby providing empirical evidence for constructing multidimensional OF intervention strategies that integrate contextual, psychological, and physiological dimensions.

## 2 Method

### 2.1 Study design

This study adopted a multi-center cross-sectional design. From October 2024 to May 2025, five medical institutions (including 2 community hospitals and 3 regional medical centers) in Jiangsu Province were selected to recruit eligible older hypertensive adults who were inpatients for continuous enrollment. All study participants signed informed consent forms.

### 2.2 Participants

Inclusion criteria: (1) Age ≥60 years; (2) Diagnosed according to the “*ISH 2020 International Hypertension Practice Guidelines*” (23) with a disease duration ≥1 year; (3) At least 4 natural teeth retained (excluding the third molar); (4) Complete clinical data of the patient.

Exclusion criteria: (1) Secondary hypertension; (2) Inability to eat orally; (3) Terminal malignant tumors; (4) Patients with end-stage severe diseases, such as those affecting the brain, heart, lungs, kidneys, or liver, and a life expectancy ≤3 months; (5) Critically ill patients in the acute phase; (6) Patients with oral diseases caused by trauma; (7) Cognitive impairment, screened using the Short Portable Mental Status Questionnaire (SPMSQ) with a cutoff of ≥ 3 errors (24); (8) Patients unable to cooperate with the questionnaire due to sensory impairments (e.g., hearing or visual impairments).

### 2.3 Sample size

This study adopted the calculation method based on sample size formulas from epidemiological cross-sectional studies (25):


$$n=\frac{Z_{1-\alpha /2}^{2}\times P(1-P)}{\delta^{2}}$$

The hypothesis testing level *α* was set at 0.05, with the corresponding Z-score (*Z*_1-a/2_) of 1.96, and the allowable error *δ* was set at 5%. Literature review revealed (26) that 41.5% of older hypertensive adults exhibited symptoms of OF, resulting in a calculated minimum sample size of *n* = 373. A total of 776 questionnaires were collected, of which 36 were excluded as invalid due to logical contradictions or incomplete responses, leaving 740 valid questionnaires for final analysis, corresponding to a response rate of 95.36%. The patient enrollment process is detailed in Supplementary Material S1. The mean age of the patients was 70.71 ± 6.72 years, ranging from 60 to 89 years. Among them, 393 were male (53.11%) and 347 were female (46.89%).

### 2.4 Data collection and quality control

This study was conducted in accordance with the STROBE guidelines (27). In August 2024, the researchers conducted a pilot study on 20 older hypertensive adults at the Department of Cardiology, Liuhe District People’s Hospital. The results of the pilot study showed that: the reliability of the SMSPH, OFI-8, GAD-7, and MNA-SF scales was good; the questionnaire was operable, with an average completion time of 15 min, which was within the participants’ acceptable range. Based on the results of the pilot study and patients’ feedback, the researchers revised the questionnaire and the study protocol.

Prior to the start of the study, standardized training was uniformly provided to questionnaire investigators and dental examiners to ensure consistency in terminology usage and assessment criteria. On the day after a participant’s admission, the investigators initially screened age-eligible patients through the electronic medical record system, and then further evaluated the inclusion and exclusion criteria in the ward. After obtaining informed consent, the investigators collected questionnaire data through face-to-face, one-on-one personal interviews in the ward. During the interview, the investigator read each question aloud and objectively recorded the participant’s answers on the questionnaire. At the end of the survey, we excluded questionnaires with incomplete answers.

### 2.5 Measurements

#### 2.5.1 Assessment of hypertension self-management ability

This study employed the self-management scale for patients with hypertension (SMSPH) initially developed by Liu et al. (28), to measure the HSMA of participants. The scale assesses the overall self-management capability across four dimensions: pharmacy, exercise, living habits, and risk factor management. A higher score is associated with patients’ higher capability to perform self-management. The scale has a Cronbach’s *α* coefficient of 0.854 and a content validity index of 0.976.

#### 2.5.2 Assessment of anxiety

To assess anxiety symptoms, the Generalized Anxiety Disorder7-item Scale (GAD-7), developed by Spitzer et al., was employed. This scale serves as a simple and effective screening tool for anxiety, demonstrating good validity and reliability in identifying and grading anxiety patients (29). It consists of 7 items scored using a 4-point Likert scale. Higher scores indicate more severe anxiety. The scale ranges from 0 to 4 for no anxiety, 5 to 9 for mild anxiety, 10 to 13 for moderate anxiety, and 14 to 21 for severe anxiety. This scale demonstrates good consistency among elderly Chinese population, with a Cronbach’s *α* coefficient of 0.84 (21).

#### 2.5.3 Assessment of nutritional status

Nutritional status was assessed using the Short-Form Mini-Nutritional Assessment (MNA-SF) developed by Rubenstein (30). It encompasses six items in total, including the body mass index (BMI) or calf circumference (0 ~ 3 points), recent weight change (0 ~ 3 points), acute disease or major psychological changes (0 ~ 2 points), mobility (0 ~ 2 points), neuropsychiatric diseases (0 ~ 2 points) and appetite (0 ~ 2 points). The scores range from 0 to 14 points, in which a score ≥11 points is considered normal nutritional status, whereas a score < 11 points indicates risk of malnutrition. The Cronbach’s *α* is 0.711 for this measure (31).

#### 2.5.4 Assessment of oral frailty

In this study, the Oral Frailty Index-8 (OFI-8) developed by the Japanese Dental Association was used to assess the degree of oral frailty in the elderly population (32), with a Cronbach’s α of 0.949. The questionnaire consists of 8 questions: (1) Do you have any difficulty eating hard foods compared to 6 months ago? (2) Have you recently choked while drinking tea or soup? (3) Do you use dentures? (4) Do you frequently experience dry mouth? (5) Are you less likely to go out than last year? (6) Can you eat hard foods such as dried squid or pickled radish? (7) How many times do you brush your teeth per day? (3 times or more/day) and (8) Do you visit a dentist at least once a year? The scoring range is from 0 to 11, with higher scores indicating poorer oral health status. The questionnaire has been validated for use among the Chinese population and has demonstrated excellent reliability and validity (33).

#### 2.5.5 Other covariates

The general data survey form for the study subjects covered four major observation dimensions: sociodemographic characteristics (age, gender, marital status, place of residence, BMI, drinking history, smoking history), socioeconomic dimensions (educational level, housing arrangement, pre-retirement occupation), disease-related factors (comorbidity index, duration of hypertension, Barthel index), and laboratory indicators (serum albumin, total protein, creatinine, platelets, serum calcium). To evaluate these factors, this study independently designed a structured questionnaire through literature review (refer to the Supplementary Material S2).

### 2.6 Statistical analysis

Statistical analysis was performed using SPSS 26.0, AMOS 28.0, and Empower Stats 3.0. Participants with missing data on the scale were excluded from the analysis, and no data imputation method was used. A rigorous data screening process was implemented prior to the main analysis: first, Harman’s single-factor test was employed to examine common method bias; subsequently, the variance inflation factor (VIF) was calculated to detect multicollinearity among independent variables. In the descriptive analysis, after testing the normality of continuous variables using the Kolmogorov–Smirnov test, normally distributed data were presented as means ± standard deviation (SD) and compared between groups using the independent samples t-test. Non-normally distributed data were expressed as medians (IQR) and analyzed using the Mann–Whitney U test. Categorical data were reported as *n* (%) and compared using the chi-square test. Based on the literature, potential covariates were predetermined, and variables that were significantly associated with OF were identified through univariate analysis and retained as control variables. Spearman’s correlation analysis was performed to evaluate the relationships among the core variables. A chain mediation model was established using AMOS 28.0, with control variables included by the forced entry method. The bias-corrected Bootstrap method with 5,000 resamples was used to estimate the 95% confidence intervals for the mediating effects. Finally, a generalized additive model was constructed to fit the nonlinear relationship between HSMA and OF using a cubic smoothing spline.

### 2.7 Ethical considerations

This study was conducted in accordance with the Declaration of Helsinki and was approved by the Ethics Committee of Yangzhou University Luhe Clinical Medical College (Approval No.: LHLL2024037), the Ethics Committee of Xinhuan Community Health Service Center (Approval No.: XHLL202501), the Ethics Committee of Donggou Community Health Service Center (Approval No.: DGLL202501), the Ethics Committee of Nanjing Hospital Affiliated to Nanjing Medical University (Approval No.: KY20250519-KS-01), and the Ethics Committee of Jiangsu Provincial People’s Hospital (Approval No.: 2025-SRFA-462). All participants provided written informed consent prior to participation. No compensation was provided to participants in this study. The research protocol was designed to ensure the protection of participants’ privacy and the confidentiality of the collected data.

## 3 Results

### 3.1 Common method bias

The data in this study were collected through self-reported questionnaires, which may introduce common method bias. To mitigate this, we implemented necessary controls during measurement, including protecting participant anonymity, explicitly stating in informed consent forms that the data would be used solely for scientific research, and applying reverse scoring to certain questionnaire items. Additionally, we conducted Harman’s one-factor factor analysis for statistical control prior to data analysis, followed by non-rotated principal component factor analysis for all variables. The results revealed eight factors with eigen values greater than 1, among which the first factor accounted for only 13.13% of the variance, significantly below the 40% threshold (34). This indicates that the study does not exhibit substantial common method bias. Finally, multicollinearity among predictor variables was evaluated using VIFs. All VIF values were substantially below the recommended cutoff of 5, indicating that collinearity was not a concern and the predictors were sufficiently independent for further analysis (35).

### 3.2 Baseline characteristics

Table 1 showed that patients with OF had older age, heavier comorbidity burden, poorer activities of daily living capacity, and lower serum albumin levels compared to those without oral frailty. Additionally, there was a significant difference in educational level between the two groups. The positive outcome rate was 47.87% (*n* = 355/740).

**Table 1:** Characteristics of the study participants grouped by oral frailty (OF).

Characteristics	Categories	Non OF (*N* = 385)	OF (*N* = 355)	*p*
Sociodemographic features				
Age, year		69.00 (63.00,73.00)	73.00 (68.00, 77.00)	**<0.001**
BMI, kg/m^2^		24.75 ± 3.09	24.53 ± 3.47	0.351
Gender, *N* (%)				0.374
	Male	211 (54.8%)	182 (51.3%)	
	Female	174 (45.2%)	173 (48.7%)	
Marital status, *N* (%)				0.059
	Married	311 (80.8%)	267 (75.2%)	
	Unmarried	5 (1.3%)	9 (2.5%)	
	Divorced	3 (0.8%)	0 (0.0%)	
	Widowed	66 (17.1%)	79 (22.3%)	
Smoking, *N* (%)				0.17
	No	234 (60.8%)	234 (65.9%)	
	Yes	151 (39.2%)	121 (34.1%)	
Drinking, *N* (%)				0.121
	No	245 (63.6%)	246 (69.3%)	
	Yes	140 (36.4%)	109 (30.7%)	
Socioeconomic dimension				
Barthel index		3.00 (3.00, 4.00)	3.00 (3.00, 4.00)	**0.003**
Educational level, *N* (%)				**0.016**
	Illiterate	119 (30.9%)	146 (41.1%)	
	Primary school	107 (27.8%)	95 (26.8%)	
	Junior high school	82 (21.3%)	64 (18.0%)	
	Senior high school	58 (15.1%)	43 (12.1%)	
	Above senior high school	19 (4.9%)	7 (2.0%)	
Residence, *N* (%)				0.154
	Rural	221 (57.4%)	223 (62.8%)	
	Urban	164 (42.6%)	132 (37.2%)	
OBR, *N* (%)				0.057
	Farmer	145 (37.7%)	160 (45.1%)	
	Worker	172 (44.7%)	154 (43.4%)	
	Freelancer	50 (13.0%)	28 (7.9%)	
	Other	18 (4.7%)	13 (3.7%)	
Dietary preference, *N* (%)				0.440
	No preference	72 (18.7%)	74 (20.8%)	
	Light taste	216 (56.1%)	190 (53.5%)	
	Sweet	18 (4.7%)	24 (6.8%)	
	Salty	53 (13.8%)	51 (14.4%)	
	Spicy	26 (6.8%)	16 (4.5%)	
Diet type, *N* (%)				0.582
	Predominantly meat	19 (4.9%)	21 (5.9%)	
	Predominantly vegetable	101 (26.2%)	102 (28.7%)	
	Balanced diet	265 (68.8%)	232 (65.4%)	
Clinical features				
ACCI		2.00 (1.00,2.00)	2.00 (2.00,3.00)	**<0.001**
SMSPH		90.00 (85.00,95.00)	87.00 (82.00,92.00)	**<0.001**
MNA-SF		12.00 (11.00,13.00)	11.00 (10.00,12.00)	**<0.001**
Duration of Illness, *N* (%)				0.277
	1–3 years	80 (20.78%)	66 (18.59%)	
	4–9 years	76 (19.74%)	87 (24.51%)	
	≥10 years	229 (59.48%)	202 (56.90%)	
Gingival bleeding, *N* (%)				0.687
	No	342 (88.8%)	311 (87.6%)	
	Yes	43 (11.2%)	44 (12.4%)	
Anxiety, *N* (%)				**<0.001**
	Minimal anxiety	331 (86.0%)	212 (59.7%)	
	Mild anxiety	46 (11.9%)	121 (34.1%)	
	Moderate anxiety	7 (1.8%)	21 (5.9%)	
	Severe anxiety	1 (0.3%)	1 (0.3%)	
Laboratory indicators				
Serum albumin, g/L		39.92 ± 3.96	38.92 ± 4.00	**<0.001**
Creatinine, μmol/L		71.50 (59.30,85.00)	72.3 (60.43,85.98)	0.370
Total protein, g/L		63.90 ± 6.08	63.58 ± 6.21	0.481
Platelet,×10^9^/L		187.00 (154.00,222.00)	180.00 (152.00,224.00)	0.977
Serum calcium, mmol/L		2.27 ± 0.12	2.27 ± 0.12	0.804

ACCI, age-adjusted charlson comorbidity index; BMI, body mass Index; OBR, occupation before retirement; SMSPH, Self-management scale for patients with hypertension; MNA-SF, short form mini nutritional assessment; p, *p*-value; IQR, Interquartile Range.

1. Data are presented as Mean ± Standard Deviation, Median (Interquartile Range), or Number (%).

2. Due to rounding, percentages may not sum to 100%.

3. Significant values are given in bold.

### 3.3 Correlational analyses of the main variables

Spearman’s correlation analysis revealed that HSMA was negatively correlated with OF (*r* = −0.310, *p* < 0.001); Anxiety was positively correlated with OF (*r* = 0.442, *p* < 0.001); and nutritional status was negatively correlated with OF (*r* = −0.400, *p* < 0.001). Additionally, HSMA was negatively correlated with anxiety (*r* = −0.292, *p* < 0.001) and positively correlated with nutritional status (*r* = 0.297, *p* < 0.001); whereas anxiety was negatively correlated with nutritional status (*r* = −0.305, *p* < 0.001). Detailed results are presented in Table 2.

**Table 2:** Descriptive statistics and correlations among key study variables (*N* = 740).

Variables	*M*	*SD*	1	2	3	4
Oral frailty	3.519	2.153	1			
Self-management ability	87.839	7.698	−0.319***	1		
Nutritional status	12.000	1.840	−0.400***	0.297***	1	
Anxiety	2.935	3.256	0.442***	−0.292***	−0.305***	1

1. Data are presented as mean and standard deviation; correlation coefficients are Spearman’s *r* values.

2. *** *p* < 0.001.

### 3.4 Mediation analysis

Correlation analysis revealed significant correlations among HSMA, anxiety, nutritional status, and OF, which met the statistical criteria for further mediation analysis. In this study, HSMA was set as the independent variable, OF as the dependent variable, and anxiety and nutritional status as mediating variables. Control variables were entered into the model using the forced-entry method. All statistically significant variables from the univariate analysis, such as age, education level, ACCI, serum albumin, and Barthel index, were simultaneously included to control for confounding effects and avoid variable selection bias. All continuous variables retained their original units to ensure clinical interpretability, and categorical variables were included as ordinal variables.

The fit indices of the mediation model indicated excellent model fit: Chi-square freedom ratio (CMIN/DF) = 2.796, root mean square error of approximation (RMSEA) = 0.049, comparative fit index (CFI) = 0.953, goodness of fit index (GFI) = 0.936, and Tucker-Lewis index (TLI) = 0.942.

As illustrated in Table 3, this study demonstrates that HSMA has a direct negative predictive effect on anxiety (*β* = −0.098, *p* < 0.001), which in turn positively predicted OF (*β* = 0.172, *p* < 0.001). Additionally, HSMA has a direct negative effect on OF (*β* = −0.037, *p* < 0.001), thus supporting (H1) and (H2). HSMA positively predicts nutritional status (*β* = 0.050, *p* < 0.001), and nutritional status negatively predicts OF (*β* = −0.166, *p* < 0.001), thus supporting (H3). Furthermore, anxiety negatively predicts nutritional status (*β* = −0.107, *p* < 0.001), thus validating (H4) via a serial mediation pathway.

**Table 3:** Intermediary path analysis (*N* = 740).

Path	*β*	S. E.	*p*	Bootstrapping 95% CI	
				Lower	Upper
Hypertension self-management ability→oral frailty	−0.037	0.009	**<0.001**	−0.054	−0.019
Hypertension self-management ability→nutritional status	0.050	0.008	**<0.001**	0.034	0.066
Nutritional status→oral frailty	−0.166	0.041	**<0.001**	−0.246	−0.086
Hypertension self-management ability→anxiety	−0.098	0.015	**<0.001**	−0.127	−0.068
Anxiety→oral frailty	0.172	0.022	**<0.001**	0.128	0.215
Anxiety→nutritional status	−0.107	0.020	**<0.001**	−0.146	−0.068

β, standardized path coefficient; S. E., standard error; CI, confidence interval; p, *p*-value.

1. Path coefficients are derived from structural equation modeling.

2. Bootstrapping was performed with 5,000 resamples; statistically significant paths are indicated by bold *p* values.

**Figure 1:**
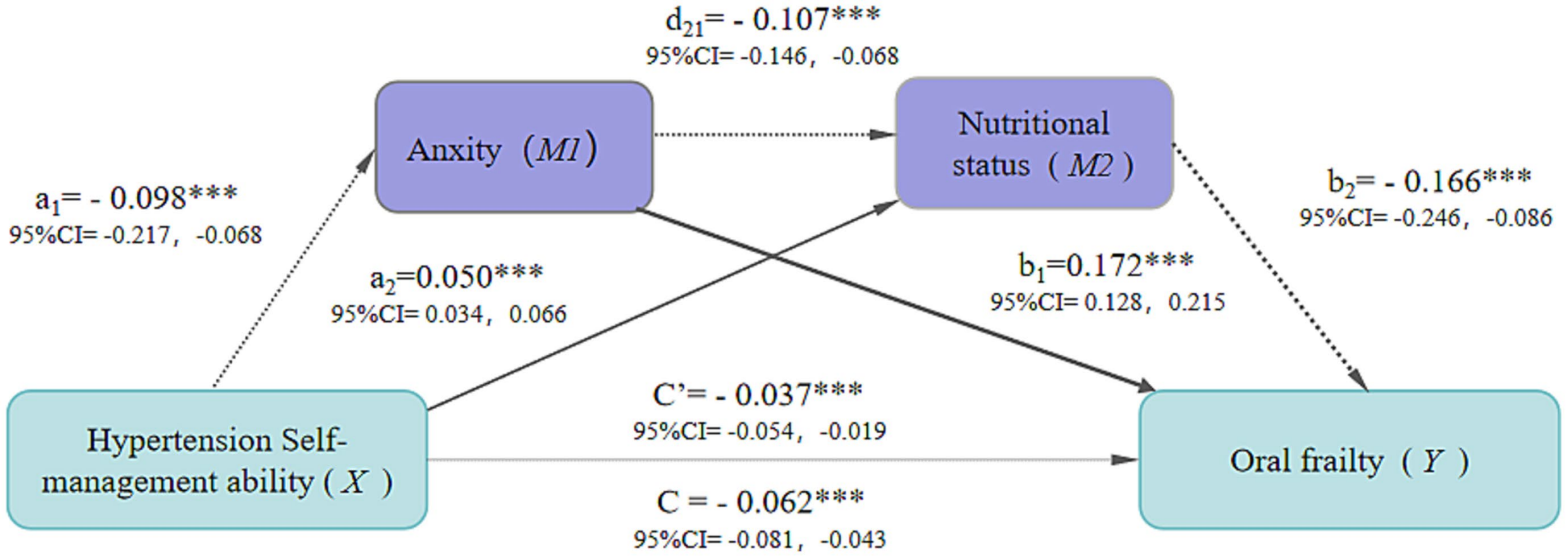
A serial mediation model of the relationship between hypertension self-management ability and oral frailty. Association of hypertension self-management ability (*X*) and oral frailty (*Y*) mediated by anxiety (*M1*) and nutritional status (*M2*). a_1_ represents the effect of *M1* on *X*; a_2_ represents the effect of *M*2 on X; b_1_ represents the effect of Y on M1; b2 represents the effect of *Y* on *M2*; d_21_ represents the effect of *M2* on *M1*; C′ represents the direct effect, and C represents the total effect. Solid lines indicate positive effects (*β* > 0), and dashed lines indicate negative effects (*β* < 0). All paths are statistically significant (*** *p* < 0.001). The model was adjusted for age, education level, serum albumin, Barthel index, and ACCI.

The mediation model was tested using Hayes’ proposed method (36) with Bootstrap analysis. The 95% CIs of the total effect, direct effect and indirect effects did not contain zero. Results (Table 4 and Figure 1) illustrate the path analysis findings for all variables. The path standardized coefficients (*β*) showed that all relationships in the model were significantly correlated. The direct effect from HSMA to OF was −0.037, with a total mediation effect of −0.034. This mediating effect is associated with three pathways: mediation via anxiety (*β* = −0.017, 95%CI –0.024 to −0.011) which accounts for 23.9% of the total effect, mediation via nutritional status (*β* = −0.008, 95%CI –0.014 to −0.004) which accounts for 11.3% of the total effect, and serial mediation via both anxiety and nutrition (*β* = −0.009, 95%CI –0.019 to −0.003), which accounts for 12.7% of the total effect.

**Table 4:** The total, direct and indirect effects revealed by the SEM analysis.

Model pathway	*β*	S. E.	Bootstrapping 95% CI		Mediating effect
			Lower	Upper	
Total effect					
Hypertension self-management ability →oral frailty	−0.062	0.010	−0.081	−0.043	100%
Direct effect					
Hypertension Self-management ability →oral frailty	−0.037	0.009	−0.054	−0.019	52.10%
Total indirect effect	−0.034	0.004	−0.042	−0.026	47.90%
Indirect effect via anxiety	−0.017	0.003	−0.024	−0.011	23.90%
Indirect effect via nutritional status	−0.008	0.003	−0.014	−0.004	11.30%
Indirect effect via anxiety and nutritional status	−0.009	0.004	−0.019	−0.003	12.70%

*β*, standardized path coefficient; S. E., standard error; CI, confidence interval.

### 3.5 Generalized additive model

**Figure 2:**
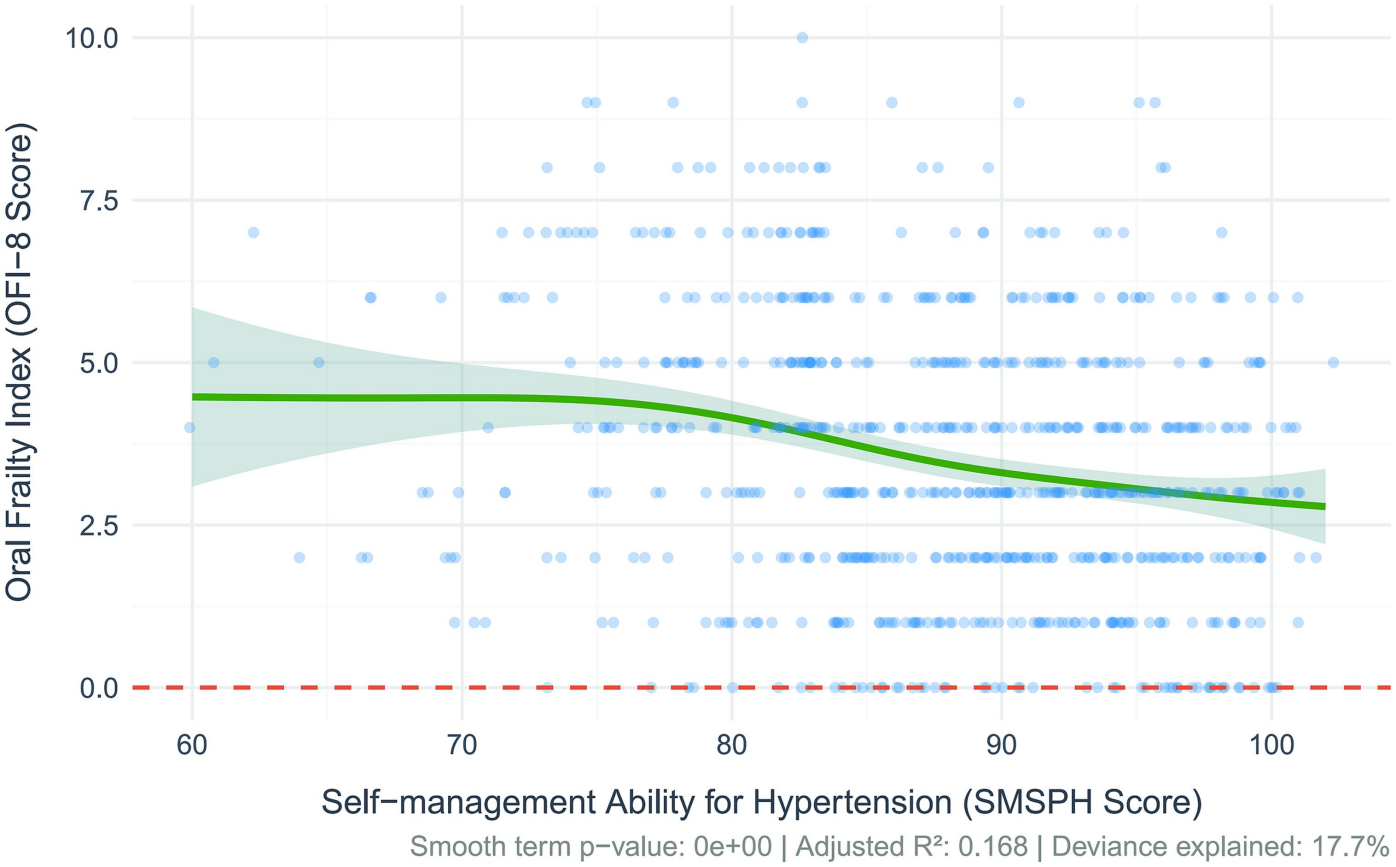
Non-linear relationship between hypertension self-management ability and oral frailty: A generalized additive model analysis. The solid line represents the smoothed fit, and the shaded area indicates the 95% confidence interval. The model is adjusted for age, educational level, serum albumin, BI, and ACCI. Smooth term *p* < 0.001, adjusted *R*^2^ = 0.168, deviance explained = 17.7%.

This study employed the smoothing curve fitting module of EmpowerStats software (37) to investigate the nonlinear relationship between SMSPH and OFI-8. The cubic smoothing spline model resulted in an effective degree of freedom (edf) of 3.68 for the smoothing term, indicating a substantial nonlinear trend. Also, the goodness-of-fit R^2^ of the model was 0.168, the bias explanation rate was 17.7%, and the smoothing term *p* < 0.001, confirming a statistically significant nonlinear association between the two variables. When the SMSPH score was below 85, OFI-8 showed a downward trend with the increase in SMSPH. When SMSPH exceeded 90, the decrease in OFI-8 slowed significantly, and the improvement effect tended to stabilize Figure 2.

## 4 Discussion

Building on TOUS, this study systematically elucidates the multi-path and nonlinear mechanisms by which HSMA affects OF in hypertensive patients. Empirical analysis demonstrates that HSMA not only exerts a direct negative impact on oral frailty but also indirectly influences it through two mediating pathways: anxiety and nutritional status. The total mediating effect accounted for 47.90% of the total effect, revealing that our mediators were crucial in explaining the association between HSMA and OF.

### 4.1 Direct association between hypertension self-management ability and oral frailty

The direct effect of HSMA on OF accounts for 52.1% of the total effect, indicating that lower levels of self-management ability are associated with a higher risk of oral frailty among older hypertensive adults, which supports (H1). Developing effective hypertension self-management habits can significantly reduce blood pressure (38). Physiologically, controlled blood pressure improves systemic microcirculation, enhancing blood perfusion and the health of local oral tissues. In addition, individuals with higher self-management ability often internalize healthy lifestyle practices—such as smoking cessation, alcohol moderation, and reduced consumption of coffee and strong tea—as stable habits. These practices naturally extend to oral health, manifesting as more regular oral hygiene practices, higher compliance with dental visits, and regulation of the oral microbiota through physical exercise (23), creating cross-domain health synergies. However, this positive impact of self-management ability does not exhibit a continuous linear progression. When the SMSPH score is below 85, improvements in self-management ability have a more pronounced effect on alleviating OF. This nonlinear relationship provides clear guidance for public health resource allocation, suggesting that future interventions should shift from “universal” ability enhancement to “precision-targeted” resource deployment. Specifically, prioritizing targeted reinforcement interventions for populations with weak self-management ability can achieve overall improvements in oral health with greater cost-effectiveness, thereby maximizing the clinical value and social benefits of health interventions.

### 4.2 The mediator role of anxiety

This study confirms that anxiety serves as a significant psychological mediator linking self-management ability and OF, accounting for 23.9% of the total effect, which supports our (H2). Previous research has demonstrated (11) that HSMA is positively correlated with psychological resilience, and more abundant psychological support can reduce feelings of helplessness, which is of great significance in alleviating anxiety (39). At the mechanistic level, anxiety affects oral health through dual pathways. From a physiological perspective, anxiety activates the hypothalamic–pituitary–adrenal (HPA) axis, leading to elevated cortisol levels, which directly suppresses local oral immune function (40). From a behavioral perspective, anxiety induces emotional eating and weakens self-management motivation (41), manifested as an increased immediate gratification need for high-sugar foods and a systematic decline in oral hygiene adherence. This anxiety-driven behavioral pattern collectively forms a dual damage pathway to nutritional intake and oral hygiene maintenance, ultimately resulting in substantial deterioration of dental health. Notably, a systematic review revealed that relaxation interventions significantly reduce both blood pressure and anxiety levels (42). Therefore, effectively incorporating relaxation training into comprehensive hypertension management not only benefits mental health but may also produce a synergistic protective effect on oral health by blocking the aforementioned mechanisms.

### 4.3 The mediator role of nutrition

Additionally, this study found that nutritional status mediates the relationship between HSMA and oral function (OF), with a mediation effect size of 11.3%, which is lower than the mediation effect of anxiety (22.4%) and the chain mediation effect (15.5%), validating our Hypothesis 3 (H3). This relatively weak mediation effect may stem from the baseline nutritional characteristics of the study subjects. As shown in Table 2, the average MNA-SF score of the participants was 12 (standard deviation = 1.84), indicating that the overall nutritional status of this population is within the normal range. In populations with adequate nutrition, the mediating role of nutritional status in the relationship between hypertension self-management and oral frailty was not statistically significant. Notably, despite the weak mediation effect, hypertension self-management is still significantly correlated with nutritional status, which is consistent with previous research findings. Prior studies (16) using the MNA-SF score and serum albumin levels revealed that the severity of OF is independently associated with malnutrition indicators. Building on this, our study confirmed the mediation effect even after controlling for key nutritional indicators such as serum albumin, thereby enhancing the reliability of the results.

These findings have important clinical implications. On the one hand, for elderly hypertensive patients with normal nutritional status, simple interventions targeting nutritional status may have limited effects on improving oral frailty; in contrast, a comprehensive strategy integrating psychological interventions may be more effective. On the other hand, balanced nutrient intake should still not be ignored. According to expert consensus (43), for hypertensive patients, the DASH diet model (44) is recommended, which can effectively increase the intake of potassium, calcium, magnesium, and dietary fiber. Deficiencies in magnesium and zinc may contribute to anxiety, and supplementing these nutrients can help alleviate such symptoms (45). For fat management, strictly limit saturated fatty acids and transfatty acids, and supplement with moderate amounts of nuts to maintain vascular endothelial function (46). This not only helps assist in blood pressure control (47) but also maintains the integrity of oral tissue structure by increasing blood perfusion of oral mucosa and masticatory muscles, thereby providing support for the prevention of OF at the level of nutritional intervention.

### 4.4 The chain mediating role of anxiety and nutrition

Finally, this study also found that anxiety and nutritional status play a chain mediating role between HSMA and OF, which supports (H4). Previous studies have shown that anxiety activates the hypothalamic–pituitary–adrenal (HPA) axis which increases cortisol levels, suppresses gastrointestinal motility, and causes loss of appetite, leading to insufficient intake of key nutrients such as protein, calcium, and antioxidants. This induces a pro-inflammatory mechanism that can trigger periodontitis and exacerbate the progression of OF (48). The association between malnutrition and anxiety also indicates that nutritional issues often coexist with mental health problems (49). This mechanism not only aligns with the expectations of the “unpleasant symptoms” theory but also reflects the multidimensional interactive influence of “psychological, physiological, and situational” factors. Anxiety serves as a critical link between self-management ability and nutritional status. Therefore, effective interventions should not be a simple combination of multiple dimensions. Given that the chain mediating pathway of anxiety-nutritional status accounts for 12.7% of the total effect, breaking this chain reaction is of great significance for reducing the risk of oral frailty. Thus, incorporating psychological interventions such as relaxation training into comprehensive hypertension management can not only directly alleviate anxiety and assist in blood pressure reduction but also potentially improve patients’ appetite and eating behaviors by breaking the chain of “anxiety → malnutrition,” thereby enhancing the overall efficacy of preventing and controlling OF.

To accurately estimate the mediating effect, this study included age, education level, and other indicators as control variables in the model. However, previous studies have shown that older age is positively correlated with anxiety (*β* = 0.08), while education (*β* = −1.26) and nutrition (*β* = −0.33) are negatively correlated with anxiety (21). This protective effect may stem from the role of education level in shaping self-management abilities: Patients with lower education levels also have poorer self-management abilities, manifested as lower blood pressure, and are more prone to unhealthy lifestyle habits such as smoking and drinking, which exacerbate the impact on oral health (3). Based on this, these control variables may have a moderating effect on the mediating pathways: advanced age may amplify the negative impact of anxiety on nutrition, while education level may buffer this impact. Therefore, patients with advanced age and low education are particularly susceptible to the ‘anxiety → malnutrition’ pathway and should be prioritized for intervention.

## 5 Limitations

Although this study provides new insights into the mechanisms linking HSMA and OF, few limitations should be acknowledged. First, this cross-sectional study design cannot make causal inference about the observed relationships. Due to the lack of longitudinal follow-up, we were unable to capture the temporal sequence and causal relationships among HSMA, anxiety, nutritional status, and OF. Furthermore, due to time and personnel constraints, this study was conducted exclusively in Jiangsu Province, lacking large-scale regional comparisons, which limits the generalizability of the research results. In addition, questionnaire-based data collection may introduce reporting bias and recall bias. Third, in terms of variable control and model construction, the chain-of-mediation model constructed in this study was only based on existing theoretical assumptions; however, given that the factors affecting oral health are complex and multidimensional, the established model is not the only possible explanatory pathway. For example, inflammatory factors (e.g., IL-6, TNF-*α*) may play an important serial mediating role between HSMA and OF, but they were not included in the analysis of this study. In addition, some potential confounding factors failed to be effectively controlled, including history of local oral diseases (e.g., previous periodontitis, dental caries), specific types of antihypertensive drugs, and medication adherence; meanwhile, primary and secondary hypertension were not distinguished, and the differences in pathophysiological mechanisms between the two types of hypertension may have different impacts on the mediating pathways. Therefore, future studies should adopt multicenter longitudinal cohort studies. On the basis of controlling the aforementioned confounding factors, objective indicators such as inflammatory factors, oral hygiene behaviors, and drug categories should be included, and subgroup analyses should be performed by distinguishing hypertension types, so as to provide more sufficient evidence-based basis for precise clinical interventions.

## 6 Conclusion

The study results indicated that HSMA can directly affect OF, mediated by anxiety, nutritional status, and the chain effect of anxiety and nutritional status. As HSMA plays a role in all influencing pathways, it highlights the importance of developing this ability. On this basis, supplemented by personalized psychological interventions or nutritional support, the occurrence and development of oral frailty can be more effectively prevented. This finding not only provides a new theoretical basis for analyzing the formation mechanism of oral frailty in older hypertensive adults but also establishes a critical practical entry point for formulating targeted comprehensive intervention strategies.

## Data Availability

The raw data supporting the conclusions of this article will be made available by the authors, without undue reservation.
