# Supplementary material for: The impact of self-management ability on oral frailty in older adults with hypertension: the chain mediating role of anxiety and nutritional status
Source: Front Med (Lausanne). 2026 Jun 12;13:1796196. doi: 10.3389/fmed.2026.1796196 (PMC13303365; doi:10.3389/fmed.2026.1796196)
Supplement: Supplementary file 1 [file Table_1.DOCX]

**Supplementary Material**


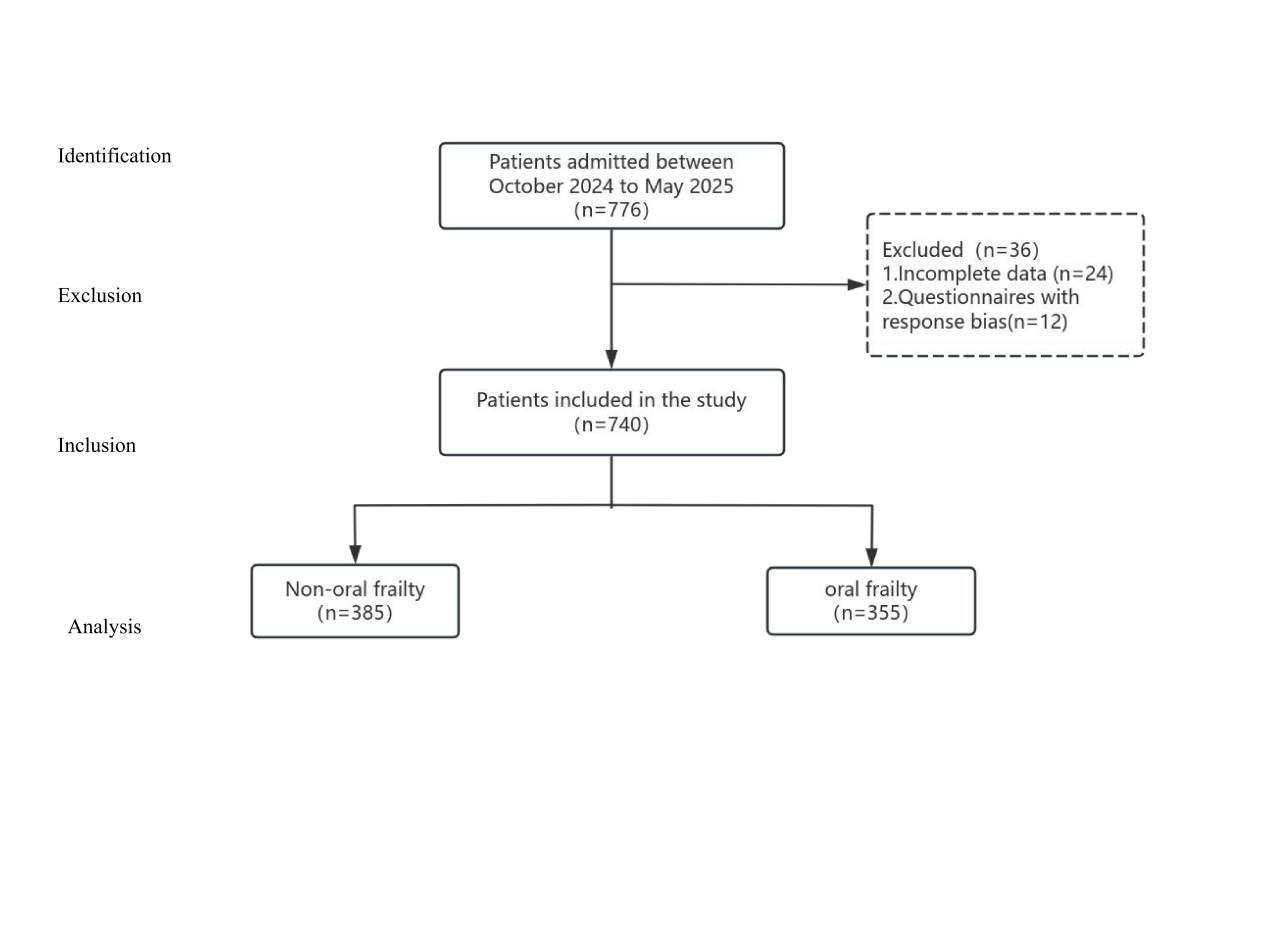
**Supplementary Material 1.** Flow chart of the screening process for the selection of eligible participants.

**Supplementary Material 2.** Structured questionnaire on oral frailty and related factors in hypertensive older adults

**Questionnaire**

**Dear Grandpa and Grandma,**

In recent years, the increasing incidence of oral diseases has not only affected the ability of patients to eat and absorb nutrients but has also caused psychological distress due to its related impacts on facial structure and appearance. Furthermore, there is a close association between oral frailty and adverse health outcomes among older adults with hypertension.

Understanding your current oral health status will enable healthcare professionals to provide more personalized advice and effective oral care measures. Therefore, we are preparing to conduct this survey, which includes two parts: an oral examination and a questionnaire, and is expected to take approximately 15–20 minutes of your time. Your participation will help us gain a more comprehensive understanding of the oral health needs of older adults.

We sincerely look forward to and welcome your participation in this survey. Please complete the questionnaire based on your personal circumstances. All information provided will be used solely for the purpose of this research analysis, and we will strictly adhere to confidentiality regulations. Please rest assured.

Thank you very much for your support and cooperation!

**1. What is your age?**

years?

1. **What is your height and weight?**

Height: ______ cm Weight: ______ kg

**3. What is your sex?**

A. Male

B. Female

**4. Do you smoke?**

A. Yes

B.NO

**5. Do you drink alcohol?**

A.Yes

B.NO

**6. What is your area of residence?**

A.Rural

B.Urban

**7. What is your marital status?**

A. Married

B. Unmarried

C.Divorced

D.Widowed

**8. What is your educational level?**

A.Illiterate

B.Primary school

C.Junior high school

D.Senior high school

E. Above senior high school

**9. What was your occupation before retirement?**

A. Workers

B. Farmer

C. Freelancer

D. Other

**10. What is your dietary preference?**

A. No preference

B. Light taste

C.Sweet

D.Salty

E.Spicy

**11. What is your diet type?**

A. Predominantly meat

B. Predominantly vegetable

C. Balanced diet

**12. What is the duration of your illness?**

A. 1-3 years

B. 4-9 years

C. ≥10 years

**13. Do you have gingival bleeding?**

A. Yes

B. No

**Thank you again for your support and cooperation.！**
